# Influence of parasite encoded inhibitors of serine peptidases in early infection of macrophages with *Leishmania major*

**DOI:** 10.1111/j.1462-5822.2008.01243.x

**Published:** 2009-01

**Authors:** Sylvain C P Eschenlauer, Marilia S Faria, Lesley S Morrison, Nicolas Bland, Flavia L Ribeiro-Gomes, George A DosReis, Graham H Coombs, Ana Paula C A Lima, Jeremy C Mottram

**Affiliations:** 1 Glasgow Biomedical Research Centre, Wellcome Centre for Molecular Parasitology and Division of Infection and Immunity, Faculty of Biomedical and Life Sciences, University of GlasgowGlasgow G12 8TA, UK; 2 Instituto de Biofisica Carlos Chagas Filho, Universidade Federal do Rio de JaneiroRio de Janeiro, RJ 21949-900, Brazil; 3 Strathclyde Institute of Pharmacy and Biomedical Sciences, University of StrathclydeGlasgow G4 0NR, UK

## Abstract

Ecotin is a potent inhibitor of family S1A serine peptidases, enzymes lacking in the protozoan parasite *Leishmania major*. Nevertheless, *L. major* has three ecotin-like genes, termed inhibitor of serine peptidase (ISP). ISP1 is expressed in vector-borne procyclic and metacyclic promastigotes, whereas ISP2 is also expressed in the mammalian amastigote stage. Recombinant ISP2 inhibited neutrophil elastase, trypsin and chymotrypsin with *K*_i_s between 7.7 and 83 nM. *L. major ISP2–ISP3* double null mutants (Δ*isp*2/3) were created. These grew normally as promastigotes, but were internalized by macrophages more efficiently than wild-type parasites due to the upregulation of phagocytosis by a mechanism dependent on serine peptidase activity. Δ*isp*2/3 promastigotes transformed to amastigotes, but failed to divide for 48 h. Intracellular multiplication of Δ*isp*2/3 was similar to wild-type parasites when serine peptidase inhibitors were present, suggesting that defective intracellular growth results from the lack of serine peptidase inhibition during promastigote uptake. Δ*isp*2/3 mutants were more infective than wild-type parasites to BALB/c mice at the early stages of infection, but became equivalent as the infection progressed. These data support the hypothesis that ISPs of *L. major* target host serine peptidases and influence the early stages of infection of the mammalian host.

## Introduction

*Leishmania* are trypanosomatid parasitic protozoa that cause a spectrum of human diseases, the leishmaniases, ranging from a lethal visceral infection to milder cutaneous ulcers. Their life cycle involves a flagellated procyclic promastigote stage that multiplies in the sandfly gut, a non-dividing flagellated metacyclic promastigote stage within the sandfly mouth parts, and a non-motile amastigote form that proliferates in mammalian macrophages. Peptidases play important roles in the pathogenicity of many parasitic protozoa, including *Leishmania* (Sajid and McKerrow, 2002; Mottram *et al*., 2004). The genome of *Leishmania major*, one of the causative agents of cutaneous leishmaniasis in the Old World, has been sequenced (Ivens *et al*., 2005), revealing the presence of at least 153 identifiable peptidases that can be classified into the major catalytic types (cysteine-, serine-, metallo-, threonine and aspartic-) and represent ∼2% of the genome. One of the major mechanisms used to control the activity of peptidases in mammals is a tight interaction with natural peptidase inhibitors such as serpins, cystatins and tissue inhibitor of metalloproteinases (Rawlings *et al*., 2004). The *L. major* genome does not appear to contain any orthologues of these molecules, yet it is not devoid of natural peptidase inhibitors (Ivens *et al*., 2005). The first to be identified in *L. major* was an inhibitor of cysteine peptidases (ICP), which is a member of the chagasin family of inhibitors first identified in *Trypanosoma cruzi* (Monteiro *et al*., 2001) and subsequently found in a variety of bacterial and protozoan pathogens (Rigden *et al*., 2002; Sanderson *et al*., 2003). In *T. cruzi* and *Trypanosoma brucei* chagasin/ICP is a modulator of parasite differentiation (Santos *et al*., 2005; 2007), while *L. major* ICP is thought to play a role in the host–parasite interaction (Besteiro *et al*., 2004). *L. major* ICP and *T. cruzi* chagasin have an unusual immunoglobulin-like fold with a cystatin-like mechanism of inhibition, which distinguishes them from all other known peptidase inhibitors (Salmon *et al*., 2006; Smith *et al*., 2006).

A second group of putative peptidase inhibitors identified in the *L. major* genome are orthologues of the *Escherichia coli* serine peptidase (SP) inhibitor ecotin and have been termed inhibitor of serine peptidases (ISPs). Ecotin is an 18 kDa protein first isolated from the periplasm of *E. coli* (Chung *et al*., 1983). It forms dimers and inhibits a wide range of SPs from the S1A peptidase family (trypsin fold) of clan PA(S), which includes trypsin, chymotrypsin, neutrophil elastase (NE) and cathepsin G (Chung *et al*., 1983; McGrath *et al*., 1995). There are no reports that ecotin inhibits SPs of other families or catalytic classes. In addition, Chung *et al*. (1983) were unable to identify an *E. coli* peptidase sensitive to ecotin, suggesting that ecotin may protect the cell against exogenous S1A peptidases (Eggers *et al*., 2004), which are involved in processes such as coagulation, fibrinolysis and host defence (Stoop and Craik, 2003).

The structure of ecotin and its mechanism of SP inhibition have been investigated extensively (McGrath *et al*., 1991a; Perona and Craik, 1997; Yang *et al*., 1998). Ecotin has also been studied as a macromolecular scaffold to develop specific peptidase inhibitors with potential therapeutic applications (Stoop and Craik, 2003). Methionine is present in its reactive site (McGrath *et al*., 1991b), although it is not essential for ecotin's inhibitory activity against trypsin (Seong *et al*., 1994). This dichotomy was resolved when the crystal structure of ecotin revealed that the protein forms a head to tail dimer that interacts with its substrate via multiple binding sites, including the active site of the peptidase (Shin *et al*., 1996; Yang *et al*., 1998). This also explains why a wide range of SPs from the S1A family are inhibited by ecotin.

Although *L. major* has 13 SPs belonging to six families, the parasite apparently lacks genes encoding SPs from the S1A family of clan PA(S) (Ivens *et al*., 2005). Three ecotin orthologous ISP genes are present in the *L. major* genome and while it is possible that the encoded ISPs could regulate the activity of *L. major* SPs other than family S1A, or those of other catalytic classes, it is likely that the ISPs, like ecotin, inhibit host SPs. This could be the trypsin and chymotrypsin-like peptidases found in the gut of the sandfly vector (Ramalho-Ortigao *et al*., 2003) or the abundance of S1A SPs that occur in the mammalian host. Postulated (*in vivo*) targets of bacterial ecotin include SPs expressed by cells of the innate immune system (i.e. neutrophils, mast cells, macrophages), such as NE, tryptase and cathepsin G, as well as enzymes participating in the coagulation cascades (Eggers *et al*., 2004). Activated neutrophils release SPs together with chromatin fibres forming extracellular traps that disarm pathogens and play a role in killing bacteria (Brinkmann *et al*., 2004). Ecotin protects *E. coli* from killing by neutrophils, primarily due to the inhibition of NE (Eggers *et al*., 2004).

Macrophages are the principle host cell for *Leishmania*, but neutrophils can act as their first residence in a mammalian host – with the infected neutrophils serving as vehicles for parasite dissemination (Laufs *et al*., 2002; Van Zandbergen *et al*., 2004; Van Zandbergen *et al*., 2007). Equally monocytes, like neutrophils express NE and cathepsin G (Kargi *et al*., 1990) and have plasma membrane receptors that enable the uptake of these enzymes from the surrounding environment (Campbell, 1982). *Leishmania* also primes mast cell degranulation following exposure to chymase and tryptase (de Oliveira *et al*., 2005). Thus we hypothesize that the numerous SPs produced and/or taken up by cells of the immune system in the course of *Leishmania* infection are potential targets for the ISPs. We begin to address the physiological targets of the *Leishmania* ISPs by creating *L. major* mutants deficient in ISP2 and ISP3 and characterizing their phenotype during the early phases of macrophage infection.

## Results

### *ISP* genes of *L. major*

**Fig. 1:**
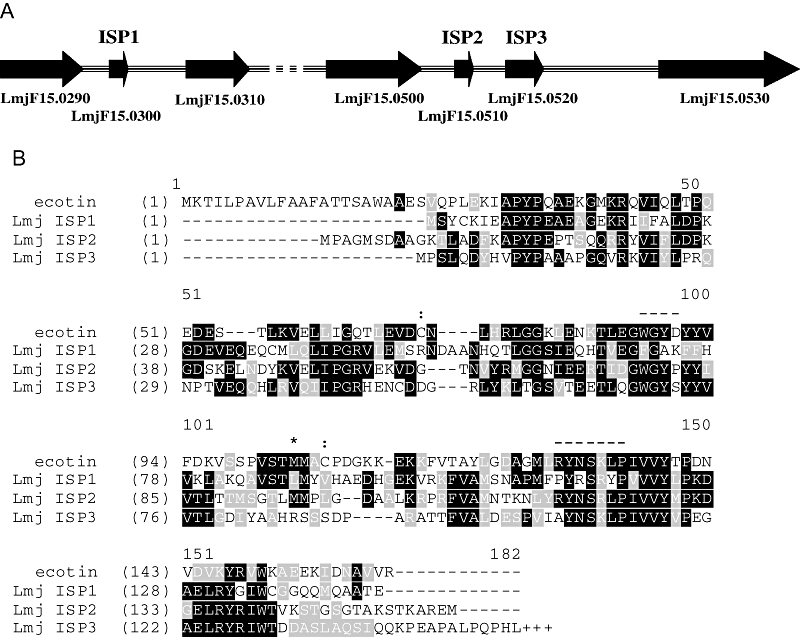
*Leishmania major ISP* genes and proteins. A. A schematic representation of the *ISP* loci of *L. major*. B. Alignment of ecotin with *L. major* ISPs. The primary P1 reactive site methionine of *E. coli* ecotin is marked by an asterisk. The two cysteine residues forming disulfide bond in the *E. coli* ecotin are highlighted above the alignment (:). The *E. coli* ecotin secondary binding site surface loops deduced from the trypsin–ecotin complex (Yang *et al*., 1998) are also highlighted (---). Identical amino acids conserved in at least two of the aligned sequences are shown in white on a black background. Similar amino acids between the aligned sequences are shown in white on a grey background. *E. coli* ecotin (GenBank CAA43954), *L. major* ISP1 (LmjF15.0300), LmjISP2 (LmjF15.0510) and LmjISP3 (LmjF15.0520). Gene identifiers from http://www.genedb.org. Truncated LmjISP3 sequence indicated by +++.

We identified three *ISP* genes in the *L. major* genome (http://www.genedb.org), *ISP1* (*LmjF15.0300*), *ISP2* (*LmjF15.0510*) and *ISP3* (*LmjF15.0520*). *L. major ISP1* is located on the same transcription unit 5′ to *ISP2* and *ISP3*, which are found in tandem (Fig. 1A). An *ISP2* homologue could be identified in the syntenic locus for both *T. brucei* (Tb927.5.1880) and *T. cruzi* (Tc00.1047053508533.40), but no *ISP3* gene was present in either of these species. *ISP1* is also present in the syntenic locus in *T. brucei* (Tb927.5.1730), but the locus could not be found in the *T. cruzi* genome – possibly because the data set is incomplete.

*Leishmania major ISP1* and *ISP2* encode predicted proteins of 16.5 kDa and 17.5 kDa respectively, which is similar to the 16.1 kDa for the mature form of *E. coli* ecotin. *ISP3* is predicted to encode a 41.8 kDa protein, with an ecotin-like domain at the N-terminal end of the protein. The C-terminal domain of the protein does not have sequence identity with known proteins or motifs. An alignment of ecotin with the three *L. major* ISPs showed that they have a shorter N-terminus compared with ecotin (Fig. 1B). *E. coli* ecotin is exported to the bacterial periplasm and the first 20 amino acids of the protein sequence act as an export signal peptide. The P1 reactive site methionine of ecotin occurs in *L. major* ISP2, but not ISP1 or ISP3 (Fig. 1B). The percentage identities between ecotin and ISP1, ISP2 and ISP3 are 32%, 32% and 30% respectively. Structural analysis of the trypsin–ecotin complex has revealed two secondary substrate-binding sites, both of which are surface loops (Yang *et al*., 1998). The primary sequence alignment of ecotin and the *L. major* ISPs shows that the amino acids of these secondary binding sites are highly conserved between the aligned sequences (Fig. 1B). However, ecotin has a disulfide bond next to its P1 methionine (Shin *et al*., 1996) and the three *L. major* ISPs lack the cysteine residues that form this bond, although they are conserved in many bacterial ecotin sequences (Eggers *et al*., 2004).

### Activity and stage-regulated expression of *L. major* ISPs

**Fig. 2:**
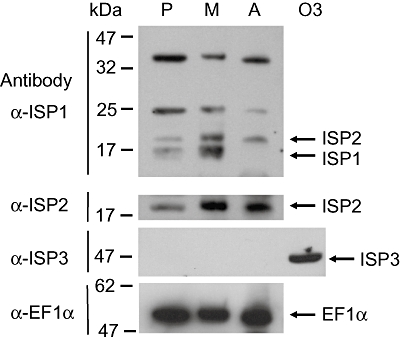
Expression of *L. major* ISPs. Western blot analyses of *L. major* cell extracts with anti-ISP antibodies. P, procyclic promastigotes at 0.5 × 10^7^ cells per lane; M, metacyclic promastigotes at 0.5 × 10^7^ cells per lane; A, lesion amastigotes at 10^7^ cells per lane; O3, promastigotes expressing ISP3 from an episome at 0.5 × 10^7^ cells per well. Elongation factor 1 alpha (EF1α) was used as a loading control.

Recombinant ISP1, ISP2 and truncated ISP3 (rISP1, rISP2, rISP3), each with an N-terminal histidine tag, were expressed and purified from *E. coli*. rISP2 was found to inhibit the human SPs, NE, trypsin and chymotrypsin, with *K*_i_s of 7.7 (±1.4) nM, 83 (±2.3) nM and 19 (±4.2) nM respectively. Antibodies raised against rISP1 and rISP2, and an antipeptide antibody raised for rISP3, allowed the expression levels of the three *L. major* ISPs to be examined by Western blot analyses of three life cycle stages (Fig. 2). The purified ISP2 antibodies did not cross-react with ISP1 but the ISP1 antibodies still recognized ISP2. However, discrete sized ISP1 and ISP2 proteins could be resolved on 4–12% gradient SDS-PAGE. Expression of ISP1 could only be detected in the procyclic and metacyclic promastigotes. ISP2 was expressed in all three life stages of the parasite with the levels of expression being greater in metacyclic promastigote and amastigote stages compared with procyclic promastigotes. ISP3 was not detected in any of the life cycle stages of the parasite. The antipeptide antibody, however, was able to detect ISP3 when it was overexpressed in procyclic promastigotes using an extra-chromosomal construct (Fig. 2). Elongation factor 1 alpha was used as a loading control.

### Generation of *L. major* mutants deficient in *ISP2* and *ISP3*

**Fig. 3:**
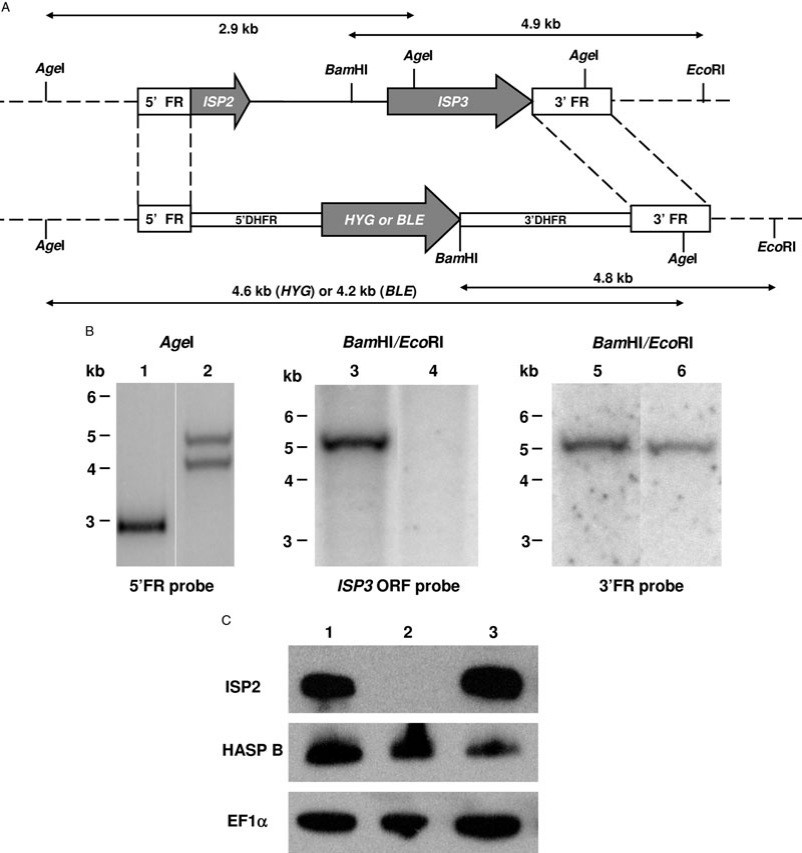
Generation of *ISP2* and *ISP3* double null mutants. A. Schematic representation of the *ISP2–ISP3* locus in WT *L. major* (upper) and the constructs for gene deletion. ORFs are shown as grey arrows and 5′ FR and 3′ FR boxes represent the FR DNA sequences used for gene targeting. The predicted DNA fragment sizes after restriction digest are shown. *HYG*, hygromycin-resistance gene; *BLE*, phleomycin-resistance gene. B. Southern blot of the WT *L. major* (lanes 1, 3 and 5) and Δ*isp2/3* (lanes 2, 4 and 6) digested with AgeI or EcoRI/BamHI and probed with radiolabelled 5′ FR, ISP3 ORF and 3′ FR probes. C. Western blot of cell extracts from 0.5 × 10^7^ purified metacyclic promastigotes of WT *L. major* (lane 1), Δ*isp2/3* (lane 2) and Δ*isp2/3 : ISP2–ISP3* (lane 3) using antibodies against ISP2, HASPB and EF1α.

An *ISP2–ISP3* null mutant (Δ*isp2/3*) was created by sequential removal of the *ISP* locus through homologous recombination using 5′ flanking region (FR) of the *ISP2* and 3′ FR of *ISP3* genes with two drug-resistance genes (Fig. 3A). Southern blotting confirmed the inactivation of both alleles, as the 2.9 kb wild-type (WT) alleles detected with a 5′ flank probe on *Age* I-digested genomic DNA (lane 1) were absent in Δ*isp2/3* (lane 2) (Fig. 3B). The absence of ISP2 in Δ*isp2/3* was confirmed by Western blotting (Fig. 3C, lane 2). As ISP3 could not be detected by Western blot analysis of WT promastigote lysates, a Southern blot using the *ISP3* open reading frame (ORF) as a probe was used to confirm that the gene had been removed from the Δ*isp2/3* genome (Fig. 3B). The complete *ISP2–ISP3* locus was re-introduced into the ribosomal locus of Δ*isp2/3* to give Δ*isp2/3 : ISP2–ISP3*. Re-expression of ISP2 in Δ*isp2/3 : ISP2–ISP3* was confirmed by Western blot analysis (Fig. 3C, lane 3), but ISP3 could not be detected using ISP3-specific antibodies and Western blotting. We observed that the levels of ISP2 expression in Δ*isp2/3 : ISP2–ISP3* is slightly higher than in WT parasites, which might result from the re-insertion of the gene in the ribosomal locus.

### ISP2 and ISP3 modulate the infectivity of *L. major* to macrophages

**Fig. 4:**
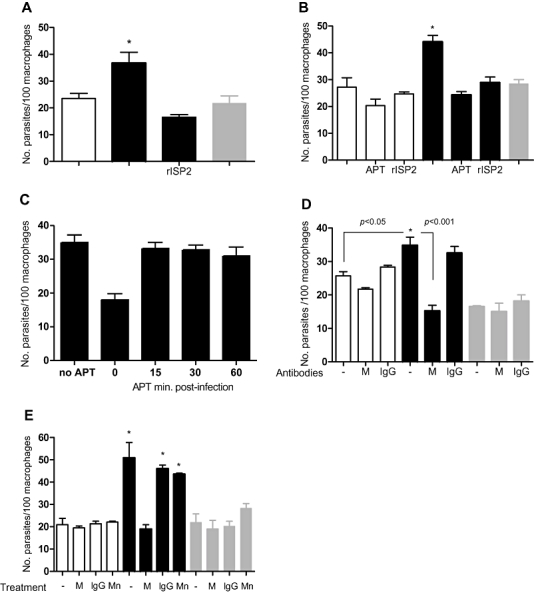
Enhanced uptake of Δ*isp2/3* by macrophages. Stationary phase promastigotes were incubated with elicited peritoneal macrophages from BALB/c mice in RPMI supplemented with 10% (v/v) FCS (A) or 0.1% (v/v) BSA (B) at a 3:1 parasite : macrophage ratio for 3 h. Extracellular parasites were removed with three washes and the adhered cells were fixed and subsequently stained with Giemsa. In (B), the interaction was performed in the presence of 4 µg ml^−1^ of purified rISP2 or aprotinin (APT). In (C), 10 µg ml^−1^ of aprotinin was incubated with macrophages for 5 min prior to incubation with Δ*isp2/3* 0, or 15, 30 or 60 min after addition of parasites and was present for the duration of the assay. In (D), macrophages were pre-incubated with 10 µg ml^−1^ of anti-CD11b M1/70 or with control rat anti-mouse IgG2b for 30 min at 37°C, followed by three washes, before addition of promastigotes. In (E) macrophages were incubated as in (D) or with 1 mg ml^−1^ of purified *Saccharomyces cerevisiae* mannan. The number of intracellular parasites was estimated by counting using light microscopy. The experiments were performed in triplicate, five independent times in A and B and two independent times in (C to E), the data are means ± SD. Key: white bars, WT parasites; black bars, Δ*isp2/3*; grey bars, Δ*isp2/3 : ISP2–ISP3*. M; anti-CD11b M1/70. In (A) the asterisk indicates statistical significance at *P* < 0.05 in relation to WT and *P* < 0.01 in relation to rISP2, in (B) the asterisk indicates significance in relation to all other samples at *P* < 0.01. In (D) the significant *p* values between samples are indicated in the graph, in (E) the asterisk indicates significance at *P* < 0.001 from all other samples without asterisk.

Δ*isp2/3* promastigotes grew *in vitro* at rates similar to those of WT or Δ*isp2/3 : ISP2–ISP3* promastigotes. The null mutants also differentiated to metacyclic promastigotes normally, as assessed by the peanut agglutinin agglutination assay (Sacks and Perkins, 1984) and the detection of the metacyclic marker HASPB (Fig. 3C). Analyses of the infectivity of stationary phase promastigotes to macrophages *in vitro* revealed that Δ*isp2/3* mutants were internalized by elicited macrophages more efficiently than WT parasites whether infections were performed in the presence (Fig. 4A) or absence (Fig. 4B) of heat-inactivated fetal bovine serum. Importantly, the increased internalization of Δ*isp2/3* was reduced to the WT levels in the presence of the SP inhibitor aprotinin or of rISP2 (Fig. 4B), indicating that the entry route used by Δ*isp2/3* is dependent on the activity of trypsin-like SPs. Neither aprotinin nor rISP2 affected the uptake of WT parasites (Fig. 4B), suggesting that the SP-dependent entry route is selectively triggered by *ISP*-deficient *L. major*. The enhanced internalization of *ISP*-deficient parasites was reverted when aprotinin was incubated with macrophages before the addition of parasites, but not if this SP inhibitor was added at later times during interaction (Fig. 4C). These observations suggest that SPs are being triggered at the initial stages of the interaction between *L. major* and the host cell. The uptake of Δ*isp2/3* by resident peritoneal macrophages was likewise higher in comparison with WT (data not shown), indicating that *ISP*2 and/or *ISP*3 modulate parasite internalization in both resting and activated macrophages.

The entry of *L. major* into macrophages is mediated in part by the interaction of the parasite with the complement 3-receptor (CR3, CD11b/CD18, Mac-1) (Mosser and Edelson, 1985; Brittingham *et al*., 1999; Kedzierski *et al*., 2004), which acts in conjunction with the mannose-fucose receptor (MFR) for parasite engulfment, at least for *L. donovani* (Blackwell *et al*., 1985). Thus we sought to address if CR3 is involved in the uptake of Δ*isp2/3* in our assay conditions, by blocking the CD11b subunit with MAB M1/70 (Fig. 4D). We observed a small reduction in the internalization of WT parasites upon CD11b blocking (Fig. 4D, white bars). However, blocking of CD11b dramatically decreased the uptake of Δ*isp2/3*, while pre-incubation of macrophages with control IgG did not affect parasite internalization (Fig. 4D, black bars). Uptake of Δ*isp2/3 : ISP2–ISP3* parasites was unaffected by CD11b blocking, strongly suggesting that enhanced uptake of Δ*isp2/3* promastigotes is mediated by increased CR3-mediated phagocytosis. Mannan purified from *Saccharomyces cerevisae* was used in order to verify if MFR was involved in parasite ingestion (Fig. 4E). As previously observed, blocking of CD11b reduced the uptake of Δ*isp2/3* but mannan did not affect the uptake of the three parasite lines, suggesting that MFR does not contribute to the ingestion of *L. major* promastigotes in this system.

### ISP2 and ISP3 modulate macrophage phagocytic activity through the inhibition of SPs

**Fig. 5:**
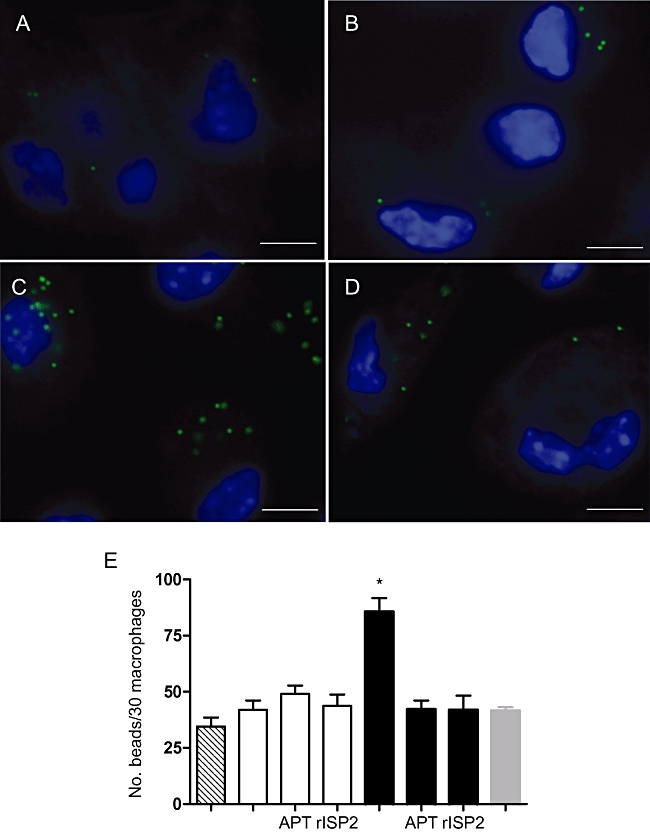
Δ*isp2/3* upregulate the phagocytic activity of macrophages by a SP-dependent mechanism. Elicited peritoneal macrophages from BALB/c mice were incubated with fluorescein-coupled latex beads in RPMI supplemented with 0.1% (v/v) BSA, for 30 min at 37°C, in the absence (A) or in the presence (B–E) of *L. major* stationary phase promastigotes at a 3:1 ratio. Extracellular beads and parasites were removed by extensive washing, the adherent cells were fixed with paraformaldehyde, DAPI-stained and observed in the fluorescence microscope. Scale bar: 1 µm. In (E), the number of intracellular beads/macrophage was estimated by counting using light microscopy and the means ± SD are presented. As indicated, aprotinin (APT) or rISP2 were added to the cultures at 4 µg ml^−1^. The experiments were performed in triplicate, three independent times. Key: hatched bar, medium; white bars, WT; black bars, Δ*isp2/3*; grey bars, Δ*isp2/3 : ISP2–ISP3*. The asterisk indicates statistical significance from all other samples at *P* < 0.05.

The entry of *L. major* into macrophages is a largely passive process for the parasite, which relies on the phagocytic capacity of host cells (reviewed in Rittig and Bogdan, 2000). Thus we examined if the overall phagocytic activity of macrophages was affected in the presence of the parasites, using fluorescein-coupled latex beads (FITC-beads) as a tracer to measure phagocytosis. The phagocytosis of FITC-beads was similar in the absence (Fig. 5A) or in the presence of WT stationary phase promastigotes (Fig. 5B), suggesting that the entry of WT *Leishmania* does not alter the basal phagocytic activity of macrophages. In contrast, the uptake of beads by macrophages increased significantly in the presence of the Δ*isp2/3* mutants (Fig. 5C) but not in the presence of the Δ*isp2/3 : ISP2–ISP3* line (Fig. 5D), indicating that exposure and/or attachment to Δ*isp2/3* selectively enhances the phagocytic activity of macrophages. Quantification of the average number of intracellular beads revealed that macrophages internalized twofold more beads in the presence of the mutants (Fig. 5E), and that the increase was reversed by addition of aprotinin or rISP2. These observations suggest that the deletion of *ISP*2 and *ISP*3 rendered *Leishmania* capable of upregulating the phagocytic activity of macrophages, resulting in more efficient uptake of the mutant parasites. Furthermore, the mechanism underlying this enhanced phagocytosis requires the activity of family S1A SP.

### ISP2 and ISP3 are required for the normal development of *L. major* in macrophages *in vitro*

**Fig. 6:**
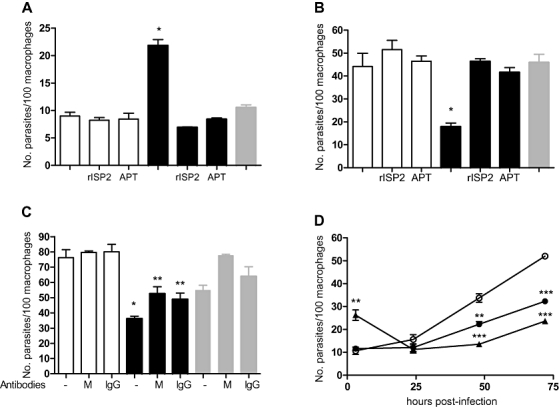
The intracellular multiplication of Δ*isp2/3* in macrophages is delayed. Stationary phase promastigotes were incubated with elicited peritoneal macrophages from BALB/c mice in RPMI supplemented with 0.1% (v/v) BSA, at a 3:1 parasite : macrophage ratio for 3 h. Extracellular parasites were removed with three washes and the adhered cells were fixed and subsequently stained with Giemsa (A) or cultured for an additional 70 h in RPMI with 10% (v/v) FCS at 37°C (B and C) before fixation and staining. The number of intracellular parasites was estimated by counting using light microscopy. In (A and B), the interaction was performed in the presence of 4 µg ml^−1^ of purified rISP2 or aprotinin (APT), where indicated. The experiments were performed in triplicate, three independent times. In (C), macrophages were pre-treated with 10 µg ml^−1^ of anti-CD11b M1/70 (M) or with rat IgG2b (IgG) for 30 min. at 37°C, followed by three washes, before addition of promastigotes. The data are means ± SD. Key: white bars, WT; black bars, Δ*isp2/3*; grey bars, Δ*isp2/3 : ISP2–ISP3*. The asterisk indicates statistical significance at *P* < 0.01 in (A) and (B). In C, the sample with an asterisk indicates statistical significance from WT and from Δ*isp2/3 : ISP2–ISP3* at *P* < 0.001 (*), and at *P* < 0.01 (**). In (D), macrophages were incubated with *L. major* for 3 h, the extracellular parasites were removed by extensive washing, the cultures were fixed at 24 h, 48 h, 72 h and Giemsa stained. The asterisks indicate statistical significance from WT parasites at *P* < 0.05 (*), *P* < 0.01 (**), *P* < 0.001 (***) at each time point. Key: circle, WT; triangle, Δ*isp2/3*; filled circle, Δ*isp2/3 : ISP2–ISP3*.

The engulfment of *Leishmania* by macrophages is followed by the *Leishmania*-containing phagosome developing into a parasitophorous vacuole, a process that involves the acquisition of some late endosomal and lysosomal characteristics (Antoine *et al*., 1998; Kima, 2007). The persistence of intracellular parasites is believed to rely on the differentiation of the engulfed promastigotes to amastigotes, which are resistant to the acidic environment and hydrolases (Kima, 2007). We examined the ability of Δ*isp2/3* to establish productive infections in macrophages *in vitro* by comparing the number of intracellular parasites soon after entry (3 h) and after a 3 day period (Fig. 6). As before, higher numbers of intracellular Δ*isp2/3* than WT were detected 3 h after infection (Fig. 6A). Also as expected, the number of intracellular WT amastigotes present at 73 h after infection (Fig. 6B) was significantly higher (44/100 macrophages) than the number of intracellular promastigotes present 3 h after infection (9/100 macrophages) (Fig. 6A). In contrast, the number of intracellular Δ*isp2/3* amastigotes (defined as amastigotes based on morphology) 73 h after infection (18/100 macrophages) was similar to that of intracellular promastigotes found 3 h after infection (22/100 macrophages) (Fig. 6B), indicating that the deletion of *ISP*2 and *ISP*3 affected the parasites’ ability to multiply inside macrophages. The number of intracellular Δ*isp2/3 : ISP2–ISP3* amastigotes was equivalent to that of WT at 73 h after infection (46/100), corroborating that the defect in parasite multiplication inside macrophages over a 3 day period was caused by the lack of ISP function. We observed release of Δ*isp2/3* parasites from infected macrophages after several days (not shown), showing that the intracellular parasites were viable.

Importantly, addition of rISP2 or aprotinin during the initial promastigote–macrophage interaction restored the ability of intracellular Δ*isp2/3* to develop normally in both elicited (Fig. 6B) and resident macrophages (data not shown). These observations suggest that the inability of intracellular Δ*isp2/*3 to multiply is a result of the lack of control of host cell SP activity at the initial stages of parasite–macrophage interaction. We asked if blocking of CR3 by M1/70 at the entry stage of Δ*isp2/3* would improve the subsequent parasite intracellular survival and/or development in macrophages (Fig. 6C), as seen for the inhibition of SPs (Fig. 6B). We found reduced numbers of intracellular Δ*isp2/3* at 3 days post infection as compared with WT and to Δ*isp2/*3 : I*SP*2/*ISP*3, even when binding to CR3 was blocked before parasite uptake, suggesting that signals triggered by CR3 are not responsible for the downmodulation of parasite growth. To analyse this more fully, we monitored the growth of intracellular parasites every 24 h after infection. This revealed that the numbers of intracellular Δ*isp2/3* had significantly declined by 24 h after infection and then multiplied only slightly up to 73 h, while WT or the re-expressing parasites increased steadily in numbers over a 3 day period (Fig. 6D). These data show that the growth of Δ*isp2/*3 parasites was delayed but not totally impaired.

Δ*isp2/3* inhibited the production of nitric oxide by lipopolysaccharide (LPS)-stimulated macrophages to the same levels as WT (not shown), removing the possibility that inability of Δ*isp2/3* to develop in the macrophage was due to defects in its capacity to induce suppression of reactive nitrogen species. Likewise, Δ*isp2/3* retained the capability to block LPS-induced expression of IL-12 by macrophages (not shown). These observations suggest that the deficient development of intracellular Δ*isp2/3* is not linked to alterations in the ability to suppress macrophage activation. Moreover, Δ*isp2/3* amastigotes isolated from mice lesions were able to infect macrophages *in vitro* and multiply at the same level as WT (data not shown), suggesting that the defect in intracellular development of Δ*isp2/3* is entirely restricted to the initial stages of infection with promastigotes.

### Infection of mice with *ISP* mutants

**Fig. 7:**
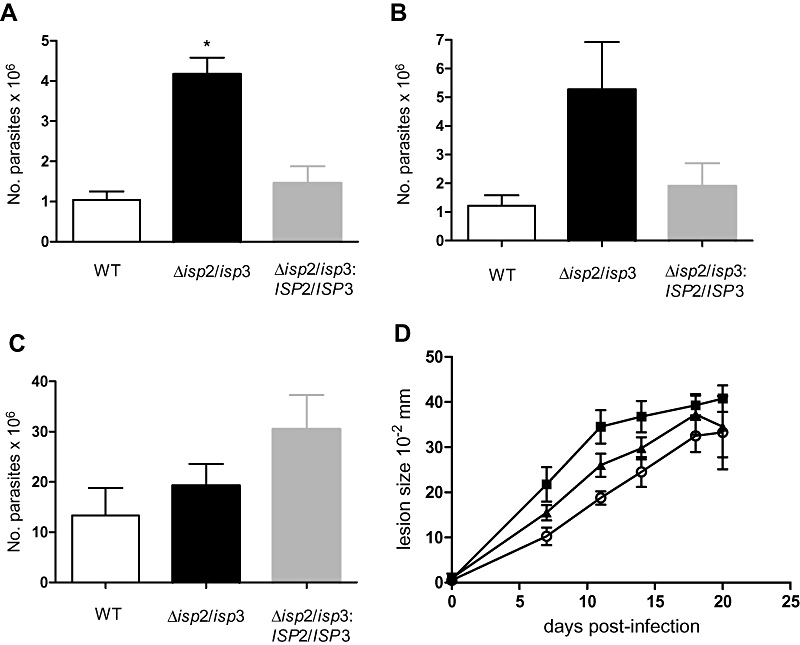
Increased growth of Δ*isp2/3* in mice at the early stages of infection. Purified metacyclic promastigotes (5 × 10^5^) were injected in the footpad of BALB/c mice (three mice per group) and the parasite burden was determined by limiting dilution *in vitro* at 24 h (A), 3 days (B) and 5 days (C) post infection. Key: white bars, WT; black bars, Δ*isp2/3*; grey bars, Δ*isp2/3 : ISP2–ISP3*. The data are means ± SD and were performed three independent times. In (A), the asterisk indicates statistical significance from WT at *P* < 0.01. In (D) BALB/c mice (five per group) were infected with metacyclic promastigotes (5 × 10^5^) in the left footpad and were injected with an equivalent volume of PBS in the right footpad. Key: square, WT; triangle, Δ*isp2/3*; circle, Δ*isp2/3 : ISP*2/*ISP*3. The experiments were performed four independent times.

The consequences of the deficiency in *ISP*2 and *ISP*3 to *Leishmania* infection in the host were evaluated by the inoculation of purified metacyclic promastigotes into the footpads of susceptible mice, followed by the determination of parasite burden for 1–5 days post infection (Fig. 7). Following 24 h of infection, the parasite burden in animals infected with the mutant line was about fourfold higher than those of mice infected with WT or the re-expressing parasites (Fig. 7A). The higher burden of Δ*isp2/3* parasites in mice was sustained until day 3 after infection (Fig. 7B), but became approximately equivalent to that of WT by day 5 (Fig. 7C). We also monitored the progression of lesions in infected mice over a 21 day period and observed that the lesions of animals infected with metacyclic promastigotes of Δ*isp2/3*, the re-expressing line or WT developed similarly (Fig. 7D).

## Discussion

In the present study we have characterized *L. major ISP* genes that encode proteins with sequence similarity to the bacterial SP inhibitor ecotin. The distribution of ecotin is unusual in that it has been found in a very limited number of bacteria; just 15 genera including *Escherichia*, *Pseudomonas*, *Salmonella and Yersinia,* from the 335 fully sequenced bacterial genomes available in the Comprehensive Microbial Resource (http://cmr.jcvi.org/cgi-bin/CMR/shared/Genomes.cgi) and in trypanosomatids (*Leishmania* and *Trypanosoma*). The *ISP* genes may therefore have arisen in trypanosomatids due to a horizontal gene transfer event from a bacterium into an organism that preceded the divergence of *Trypanosoma* and *Leishmania* (Opperdoes and Michels, 2007). There is also divergence in the trypanosomatid *ISP* gene family as *ISP3* is only found in *Leishmania* and not *Trypanosoma*. Its proximity to *ISP*2 and the unusual C terminal extension encoded indicates that *ISP3* may have arisen through an *ISP2* gene duplication event, after the divergence of *Leishmania* and *Trypanosoma*.

Ecotin is a potent inhibitor of S1A family (trypsin-fold) SPs, yet *E. coli* lacks these enzymes. This also appears to be the case for *Leishmania,* as the parasite lacks S1A family SPs but contains ISP2, which we have demonstrated to be a potent inhibitor of different mammalian S1A family SPs including trypsin and NE. In the absence of S1A family SPs in *Leishmania,* the ISPs could potentially target host trypsin-type SPs. The possible role of *Leishmania* ISPs in protecting the parasite from the digestive SPs in the sandfly gut is currently under investigation, while in this study we investigated the role of ISPs in the early stages of infection in the mammalian host. Unlike ecotin, *Leishmania* ISPs lack an N-terminal signal sequence, suggesting that they are not targeted into the secretory pathway, although they may be secreted via a non-classical secretory mechanism or released by apoptotic promastigotes (Van Zandbergen *et al*., 2006; 2007). Thus, our hypothesis incorporates the idea of inhibitor release by the parasite causing an effect on mammalian SPs.

ISP1 expression could be detected in both procyclic promastigotes and metacyclic promastigotes, but not amastigotes – consistent with the protein having a role in the sandfly vector or early in infection of the mammalian host. In contrast, ISP2 is expressed in all life cycle stages, but with a higher level of expression in metacyclic promastigotes and amastigotes, suggestive of a role in initiation and persistence of infection of the mammalian host. Here we have investigated the roles of *ISP*2 and *ISP*3 by generating *ISP*2/*ISP*3-deficient *L. major*. As expression of ISP3 was not detected in either metacyclic promastigotes or amastigotes, it is likely that the phenotypes observed in this study for Δ*isp*2/*isp*3 are primarily due to ISP2.

The phenotypic analyses of the Δ*isp*2/*isp*3 mutants showed that the lack of these genes enhanced parasite infectivity *in vitro*, as a result of increased parasite uptake by peritoneal macrophages. We also showed that exposure of macrophages to Δ*isp2/3* increased the phagocytosis of inert particles, suggesting that upregulation of macrophage phagocytic activity is responsible for increased infectivity of Δ*isp2/3 in vitro*. Increased uptake of Δ*isp2/3* was observed in the presence or absence of serum, indicating that a common mechanism susceptible to regulation by ISPs is involved. CR3 was found to be the predominant receptor used by Δ*isp2/*3, but not by WT or the re-expressing parasites. Although the fixation of complement components at the surface of *Leishmania* mainly requires the presence of serum as a source of C3 (Mosser and Edelson, 1987), the C3 endogenously produced by macrophages might represent an alternative source of complement in its absence. Additionally, LPG and gp63 have binding sites for CD11b (Russell and Wright, 1988; Talamas-Rohana *et al*., 1990). We observed that Δ*isp2/*3 adhere to macrophages more than WT at low temperature, in the absence of serum (M.S. Faria and A.P.C.A. Lima, unpubl. obs.), suggesting that more efficient adhesion contributes to increased infectivity. Although CR3-mediated phagocytosis, without FMR association, seems to be the main mechanism driving the internalization of *ISP*-deficient *Leishmania*, we cannot discount the possibility that additional receptors are involved in the uptake of the mutant lines.

Intriguingly, our results show that the receptor(s) involved in the differential engulfment of Δ*isp*2/*isp*3 are associated, either directly or indirectly, with the activity of SPs as internalization of the parasites were brought to WT levels in the presence of aprotinin or rISP2. We have not addressed which SPs are mediating increased activation of phagocytosis, but NE is one candidate. It has been reported that in polymorphonuclear leukocyte NE is an endogenous ligand for CR3, and that its binding, via an active site interaction, to CR3 modulates leucocyte adhesion via competition with other endogenous ligands (Cai and Wright, 1996). NE is also expressed by macrophages, although its association with CR3 has not been shown in this cell type. Furthermore, the activity of NE triggers toll-like receptor 4-mediated responses (Devaney *et al*., 2003), suggesting that the activity of SPs might be tightly associated with the modulation of leucocyte receptors. Another S1A family SP, factor X, has also been shown to associate with CR3, in an interaction involving peptidase activation (Altieri *et al*., 1988) and so this could also be a target of ISPs.

The binding to and activation of CR3 by *L. major* promastigotes leads to the inactivation of infected cells, contributing to the subsequent intracellular survival of the parasite and allowing amplification as intracellular amastigotes at sites of infection (Mosser and Edelson, 1987). We observed that intracellular Δ*isp2/3* were partially eliminated within 24 h of infection, and slowly recovered growth after 48 h. Importantly, Δ*isp2/3* grew normally inside macrophages when SP inhibitors were added to the initial stages of the promastigote–macrophage interaction, suggesting that the activity of SPs triggered during parasite contact is responsible for the subsequent defect in the intracellular development of engulfed parasites. The production of nitric oxide (NO) has been implicated in the control of the growth of *L. major* in mice (Wei *et al*., 1995), but we observed no difference in the capacity of Δ*isp2/3* parasites to inhibit LPS-induced NO production by macrophages as compared with WT (data not shown), suggesting that this reactive nitrogen species is not mediating selective killing of Δ*isp2/3.* However, we cannot exclude that SPs engaged by Δ*isp2/3* during interaction with macrophages trigger the production of reactive oxygen species, which could be responsible for the control of parasite growth. These observations strongly suggest that although *ISP* deletion enhances parasite entry to macrophages, this is not in fact beneficial as ISP function at the initial stages of parasite–macrophage interaction is required to prevent premature death and enable normal intracellular growth.

The defect in intracellular development of Δ*isp2/3 in vitro* was different from the result observed *in vivo*, where mice infected with Δ*isp2/3* had a higher parasite burden at the early stages of infection (up to three days) in comparison with those infected with WT or with the re-expressing lines. It has been reported that the phagocytosis of apoptotic neutrophils by macrophages infected with *L. major* enhances the intracellular multiplication of parasites in cells derived from BALB/c mice due to the induction of TGF-β (Ribeiro-Gomes *et al*., 2004). The depletion of polymorphonuclear leukocyte prior to infection resulted in lower parasite burden 13 days later, indicating that neutrophils are required for the optimal development of *L. major in vivo.* More recently, studies *in vivo* using intravital microscopy suggest that neutrophils recruited to the site of infection rapidly engulf *L. major* and are the primary host cells for the parasites within the first 48 h (Peters *et al*., 2008). *In vivo*, it is possible that, similar to what was observed in macrophage infections *in vitro*, Δ*isp2/3* could be internalized by neutrophils at higher numbers in comparison with WT parasites and survive in these cells, leading to higher parasite burden in the first 24 h. Alternatively, we could hypothesize that the signals triggered by Δ*isp2/3* during the parasite–macrophage contact, which resulted in the downregulation of intracellular parasite replication observed *in vitro*, are overcome *in vivo* by the deactivating stimulus given by neutrophils. In the absence of such downregulation, Δ*isp2/3* that were internalized at higher numbers by macrophages can develop normally, leading to enhanced parasite burden, which is controlled later on. Δ*isp2/3* was capable of generating lesions in the footpad of mice, although the lesions were slightly smaller in size in the first 11 days. The production of IFN-γ, IL-4, IL-10 and TNF-α in lymph nodes of mice 15 days after parasite inoculation was similar between infections with the different lines, suggesting that there was no major alteration in the development of the adaptive immune response. ISP1 is also expressed in metacyclic promastigotes and might compensate in part for the loss of ISP2 in the early infection process, a hypothesis that can be tested with *ISP1/ISP2/ISP3* triple mutants that we have recently generated. *ISP1* expression could not be detected in amastigotes, so is unlikely to compensate for ISP2 after establishment of infection. Thus ISPs would not appear to be required for dissemination or persistence in the mammalian host.

At the initial stages of *Leishmania* infection *in vivo*, neutrophils are the first cells recruited to the site of infection (Lima *et al*., 1998; Peters *et al*., 2008) and they have been shown to exert an important modulatory role on the outcome of the immune response in susceptible mice (Chen *et al*., 2005). In response to microbial components, neutrophils secrete the contents of azurophilic granules containing a high density of SPs, such as NE, cathepsin G and PR3, and extracellular NETs, which present trapped active NE to the surrounding milieu (Brinkmann *et al*., 2004). Therefore, it is likely that *Leishmania* are exposed to large amounts of SPs at the site of infection, which could be modulated by the ISPs.

Taken together, the results in this study suggest that the inhibition of host SPs by ISP2 at the initial stages of the parasite–macrophage interaction is required for the optimal initial intracellular adaptation of *L. major* for living in macrophages, which is crucial for the establishment of productive infections in the host. Investigations into the role of ISP in the interaction of *Leishmania* and neutrophils are ongoing and should reveal more on the role of ISPs in the infection process.

## Experimental procedures

### Parasites

*Leishmania major* Friedlin (MHOM/JL/80/Friedlin) were grown as promastigotes in modified Eagle's medium (designated HOMEM medium) supplemented with 10% heat-inactivated fetal calf serum (FCS) at 25°C as described previously (Besteiro *et al*., 2006). The following antibiotics were used at the indicated concentration for the selection of transfectants: 50 µg ml^−1^ Hygromycin B (Roche), 25 µg ml^−1^ G418 (Invitrogen), 10 µg ml^−1^ Phleomycin (InvivoGen) and 50 µg ml^−1^ Puromycin dihydrochloride (Calbiochem). *L. major* metacyclic promastigotes were isolated from stationary phase culture (2.5 × 10^7^ cells ml^−1^) by agglutination of promastigote cells with peanut lectin as described previously (Sacks *et al*., 1985). Metacyclic promastigotes represented 17.8%, 18.2% and 20.1% in *L. major* WT, Δ*isp2/3* and Δ*isp2/*3 : I*SP*2/*ISP*3 respectively. Lesion amastigotes were purified as previously described (Hart *et al*., 1981).

### Generation of *L. major ISP* null mutants, re-expressors and overexpressors

The two plasmids containing the antibiotic-resistance cassettes used for the double-allele inactivation of *ISP2*/*ISP3* were produced as follows: the 418 bp 5′ FR was generated by PCR with primers OL1403 and OL1404 (Table 1). The 600 bp 3′ FR was generated by PCR with primers OL1405 and OL1406. The PCR fragments were subcloned into pGEM-T and then released by restriction digest with HindIII/SalI for the 5′ FR, and XmaI/BglII for the 3′ FR. The fragments were sequentially cloned into similarly digested hygromycin-resistant plasmid pGL792 (Besteiro *et al*., 2004) to give pGL959. To produce a phleomycin-resistant knockout construct the hygromycin cassette from pGL959 was replaced with the SpeI/BamHI phleomycin-resistance cassette to give pGL961. The integration cassette from plasmids pGL959 and pGL961 were excised by digestion with BglII and HindIII before transfection. For the re-expression construct, a 2.8 kb PCR fragment containing ISP2 ORF, ISP3 ORF and the intergenic genomic DNA region was amplified from *L. major* DNA with the primers OL1657 and OL1473 containing BglII and NotI restriction sites. The PCR fragment was subcloned in PCRscript (Stratagene). The 2.8 kb ISP2–ISP3 insert was cloned into the pRIB expression vector (Garami and Ilg, 2001) using BglII and NotI to give pGL1005. The integration cassette from pGL1005 was excised by digestion with PacI and PmeI before transfection. *L. major* promastigotes were electroporated with 20 µg of the linearized integration cassettes and transfectants were selected with the appropriate antibiotics as previously described (Besteiro *et al*., 2006). Full-length ISP3 was amplified by PCR using primers OL1459 and OL1320. The PCR product was subcloned into pPCRscript (Stratagene). The insert was released from pPCRscript by XmaI/BamHI digest and ligated into similarly digested pXG vector generating the ISP3 overexpression construct pGL1004.

**Table 1:** Primers used in the study.

Primer	Restriction site	Sequence (restriction sites are underlined)
OL1403	HindIII	CGAAGCTTGGATCGACTCAATCACGCCAACG
OL1404	SalI	CAGTCGACTGAGTTAGAGTGGAGTGTTG
OL1405	XmaI	TACCCGGGAAGGAGCTGCACCACATGGGCA
OL1406	BglII	GCAGATCTTTGTTCATCGGAGAAGGGATGC
OL1657	BglII	GCAGATCTATGCCCGCAGGGATGTCCGAC
OL1473	NotI	ATGCGGCCGCCTACTTCCCGTCTACGGGGTC
OL1315	NdeI	CGCATATGTCATACTGCAAGATCGAGGCC
OL1316	BamHI	GCGGATCCTCACTCCGTGGCTGCCTGCATC
OL1660	NdeI	ATCATATGCCCGCAGGGATGTCCGAC
OL1318	BamHI	GCGGATCCTCACATCTCCCTTGCCTTGGTG
OL1320	BamHI	GCGGATCCCTACTTCCCGTCTACGGGGTC
OL1459	XmaI	TACCCGGGATGCCCTCCCTCCAGGACTAC

### Southern blot analysis

Genomic DNA (gDNA) was isolated from the WT and mutant cell lines with the DNeasy kit (Qiagen). Three micrograms of gDNA was digested with appropriate restriction enzymes, electrophoresed through a 0.8% agarose gel and blotted onto Hybond C Super (Amersham Pharmacia). The DNA probes were radiolabelled with a random priming kit (Stratagene) and the blots were hybridized at 65°C overnight. The membranes were washed twice with 2× SSC with 0.1% SDS for 15 min at 65°C and rinsed once with 0.2× SSC at 65°C. Phosphostorage screens were exposed overnight and the membranes visualized using a Typhoon 8610 variable mode imager (Molecular Dynamics).

### Protein expression, Western blotting and antibodies

*Leishmania major ISP1* and *ISP2* were amplified by PCR from gDNA using OL1315-OL1316 and OL1660-OL1318 respectively. The PCR products were subcloned into pPCRscript (Stratagene). The inserts were released from pPCRscript by NdeI/BamHI digest and ligated into similarly digested pET-15b (Novagen). The constructs pGL998 (ISP1/pET15b) and pGL1179 (ISP2/pET15b) were used to express N-terminal his-tagged ISP1 and ISP2 respectively. The proteins were isolated using nickel agarose affinity purification, ion-exchange chromatography. LPS was removed using Detoxi-Gel (Pierce) according to manufacturer's instructions. Purified recombinant ISP1 and ISP2 were used to raise antisera in sheep. The antibodies were purified from the serum using immobilized recombinant protein or peptide onto AminoLink Plus coupling gel (Pierce). To remove possible cross-reaction between ISP1 and ISP2 antibodies, anti-ISP1 antisera was exposed to immobilized recombinant ISP2, and vice versa, prior to purification. ISP3 antibodies were produced in rabbit against a peptide corresponding to the last 20 amino acids of the encoded protein (ASASSTKSGNGSKADPVDGK). The antibodies were purified from the antiserum using immobilized peptide onto AminoLink Plus coupling gel (Pierce).

The following *L. major* cell lysates were used for the Western blots: log phase promastigote 0.5 × 10^7^ cells per well, purified metacyclics lysates 0.5 × 10^7^ cells per well and purified lesion amastigote lysates 10^7^ cells per well. For the ISP3 Western blot, a log phase promastigote lysate overexpressing ISP3 was used (0.5 × 10^7^ cells per well). The cell lysates were separated by 4–12% gradient SDS-PAGE and the proteins were transferred onto a PVDF membrane. Affinity-purified anti-ISP1 and anti-ISP2 sheep antibodies and affinity-purified anti-ISP3 rabbit antibodies were used in Western blots to detect each individual ISP. The antibodies were used at 1/1000 dilution. A secondary anti-sheep or anti-rabbit IgG antibodies coupled to horseradish peroxidase (Santa Cruz Biotechnology) were used at a 1 in 4000 dilution. Rabbit polyclonal antiserum specific for HASPB (a gift from Prof. D. Smith, York University) was used at 1/1000 dilution. Following each Western blot, the membranes were stripped and re-probed with anti-elongation factor 1 alpha monoclonal antibody (Upstate Cell Signalling Solutions) as a loading control. This antibody was used at 1 in 5000 dilution and secondary anti-mouse IgG antibody coupled to horseradish peroxidase (Promega) was used at 1 in 4000 dilution. The Western blots were revealed by soaking the membrane in SuperSignal West Pico chemiluminescent substrate (Pierce) exposing the membranes to X-ray films.

### *K*_i_ determination

The human SPs trypsin (0.21 µM), chymotrypsin (5 nM) and NE (3 µM) were incubated in 100 mM Tris (pH 8) with varying concentrations of ISP2 (100 pM to 30 µM) for 20 min prior to the addition of the appropriate substrate, Bz-R-AMC, Suc-AAPF-AMC and MeOSuc-AAPV-AMC respectively (Calbiochem). Substrates were used at a saturating concentration, which was determined experimentally (data not shown). Activity was determined by the change in fluorescence (λ_ex_ = 355 nm, λ_em_ = 460 nm) at 21°C using an EnVision 2102 plate reader (Perkin Elmer, Bucks). The effect on the initial velocity was calculated and curves were fit using FigP (FigPsoft Corporation, Canada). Relative activity was calculated from the ratio of initial velocity in the presence of inhibitor to that of uninhibited controls. Experiments were carried out in triplicate and results are expressed as the mean ± SEM. *K*_i_ values were calculated using the equation for tight binding inhibitors in equilibrium (*K*_i_ = (*K*_i_ × [S])/([S] + *K*_m_).

### Macrophage infection assays

Peritoneal macrophages from BALB/c mice were elicited by injection of 1 ml of 1% thioglycolate in the peritoneal cavity and collected 3 days later upon injection of 5 ml of ice-cold RPMI 1640-medium (Sigma-Aldrich, Egham, UK) in the peritoneal cavity, followed by gentle massage. The cells were washed, plated onto glass coverslips in a 24-well tissue culture plate at a density of 3 × 10^5^ ml^−1^ and cultivated overnight in RPMI supplemented with 10% of FCS (Cultilab, Campinas, SP, Brazil). The cells were washed three times with Hank's balanced salt saline (HBSS) and incubated with stationary phase promastigotes in RPMI, supplemented with 0.1% bovine serum albumin (BSA) (Sigma-Aldrich, St Louis, MO, USA), at 37°C for 3 h. The coverslips were washed for the removal of extracellular parasites, fixed with Bouin overnight and stained with Giemsa (Laborclin, Pinhais, PR, Brazil). The number of intracellular parasites was determined by counting at least 100 cells per replicate under the light microscope. Where indicated in the figure legends, the infections were performed in the presence of 10% FCS. Aprotinin (Sigma-Aldrich, St., MO, USA) or rISP2 (4 µg ml^−1^ each) were added to macrophages 5 min prior to addition of the parasites and remained during the 3 h of interaction. Anti-mouse CD11b monoclonal antibodies (M1/70) or rat IgG2 (BD Bioscience Pharmingen, San Jose, CA, USA) were incubated at 10 µg ml^−1^ with macrophages in RMPI-FCS for 1 h, and removed by extensive washing before the addition of promastigotes in RPMI-BSA. For the survival assays, the macrophages were infected as described above for 3 h and after the removal of extracellular parasites the cells were cultured at 37°C, in RPMI supplemented with 10% FCS for 24, 48 or 72 h.

### Phagocytosis of latex beads by macrophages

Elicited peritoneal macrophages were plated onto glass coverslips and cultivated overnight in RPMI-10% FCS. The cells were washed with HBSS and adherent cells were incubated with Fluorescein-coupled latex beads of 500 nm diameter (Molecular Probes, USA) diluted 1:2000, in RPMI-0.1% BSA, for 30 min at 37°C. Extracellular beads were removed by washing six times with HBSS and the cells were fixed in paraformaldehyde 4%, washed with phosphate-buffered saline pH 7.2, incubated with DAPI and mounted on N-propylgalacto glass slides. The slides were observed in an Axioplan fluorescence microscope for estimation of the average number of beads per macrophage. Where indicated, *L. major* stationary phase promastigotes were co-incubated with the latex beads and the macrophages at a 3:1 parasite–host cell ratio.

### *In vivo* assay of *L. major* infectivity

Stationary phase promastigotes were centrifuged at 2000 *g*, re-suspended in 0.5 ml of HOMEM and incubated with 50 µg ml^−1^ peanut lectin for 20 min at room temperature for the agglutination of procyclic promastigotes. The cells were centrifuged at 100 *g* for 5 min, the supernatant containing metacyclic promastigotes was recovered and the cells were washed with HBSS. BALB/c mice (4–6 weeks) were infected in the footpads with 2 × 10^5^ metacyclic promastigotes and the parasite burden in paws and in the draining lymph nodes were evaluated between 1 and 10 days. The mice were sacrificed, the bottom tissue of the footpad was removed and ruptured to homogeneity with the back of a syringe through a nylon membrane in 2 ml of Schneider's medium supplemented with 10% FCS, 2% urine. The popliteal lymph nodes were collected and submitted to rupture as described above. One hundred microlitres of the cell homogenates was submitted to serial dilutions in 24-well plates and cultivated at 27°C for 5–7 days. The number of parasites in the highest dilution was estimated by counting in a Neubauer chamber. The parasite concentrations in the footpad or lymph nodes were subsequently calculated as: number of parasites/ml/well × dilution factor in 24-well plate × dilution factor of the initial homogenate.

### Statistical analyses

The analysis of significance of the data was performed by analysis of variance, using the GraphPad Prism 4.0 Program. The data were analysed by one-way anova using the Bonferroni post-test, at a significance level of 5%. In Fig. 6D the data were analysed by two-way anova using the Bonferroni post-test, at a significance level of 5%. The scores showing statistical significance are indicated in the figures with asterisks and the *P*-values are indicated in the legends.
